# Online Racial/Ethnic Discrimination, Suicidal Ideation, and Alcohol Misuse Among Ethnoracially Minoritized College Students: The Roles of Internalized Racism and Ethnic Identity

**DOI:** 10.1007/s40615-025-02381-1

**Published:** 2025-03-12

**Authors:** Lillian Polanco-Roman, Jazmin Reyes-Portillo, Chantel T. Ebrahimi, Ashley Powell, Brendesha M. Tynes

**Affiliations:** 1 The New School, Department of Psychology, 80 Fifth Avenue, Room 617, New York, NY 10011 USA; 2 Department of Psychology, Montclair State University, Montclair, NJ USA; 3 University of Southern California, Los Angeles, CA USA

**Keywords:** Online racial/ethnic discrimination, Suicidal ideation, Alcohol use, College students

## Abstract

Over the past decade, mental health problems have disproportionately increased among U.S. ethnoracially minoritized college students, though explanations for this disparity remain understudied. In the present study, we examined the direct and indirect associations between online racial/ethnic discrimination, suicidal ideation (SI), and alcohol misuse. We also investigated whether internalized racism helps explain the association between online racial/ethnic discrimination (i.e., individual or vicarious exposures) and SI and alcohol misuse. Lastly, we examined the moderating role of ethnic identity dimensions (i.e., exploration and commitment) in this indirect association. Participants included 494 ethnoracially minoritized college students ages 18–30 years (*M* = 19.62, *SD* = 2.08; 79% female; 60% Latine; 83% U.S.-born) from the U.S. who completed an online survey. Findings revealed that online racial/ethnic discrimination (i.e., individual and vicarious exposures) was directly associated with increased SI and alcohol misuse. The indirect association of online racial/ethnic discrimination (i.e., individual and vicarious) on SI via internalized racism was statistically significant. These indirect associations, however, were not observed with alcohol misuse. Ethnic identity commitment moderated the indirect association of vicarious, though not individual, online racial/ethnic discrimination on SI via internalized racism. These findings underscore the impact of online racial/ethnic discrimination on ethnoracially minoritized college students’ mental health, identify internalized racism as a potential pathway through which this type of discrimination may confer risk for SI, as well as ethnic identity commitment as a potential protective factor that may buffer these harmful effects.

**Supplementary Information:**

The online version contains supplementary material available at 10.1007/s40615-025-02381-1.

In the United States, suicide has disproportionately increased among young people from ethnoracially^1^ minoritized backgrounds [1], for whom the highest risk period of dying by suicide is before age 30 years [2]. Unsurprisingly, mental health problems including suicidal ideation (SI) have disproportionately increased among ethnoracially minoritized college students over the past decade [3]. Although ethnoracially minoritized college students report low rates of alcohol misuse, it is another common problem among all college students that has worsened over this same period including among ethnoracially minoritized students [4, 5]. One potential driver of this increase in health risk behaviors (i.e., alcohol misuse, SI) in college students that remains understudied is online experiences, and online racism-related experiences may be particularly relevant among ethnoracially minoritized college students.

## Racial/Ethnic Discrimination and Risk for Suicidal Ideation and Attempts

Racism has been identified as a social determinant of health and a principal driver of racial/ethnic disparities in health outcomes in conjunction with and independently of poverty [6]. Racism is a system of power that purposely allocates societal resources inequitably to create social conditions that disadvantage People of Color. This manifests behaviorally via experiences of racial/ethnic discrimination, or unjust treatment predicated on an individual’s racial/ethnic group affiliation [7]. Biopsychosocial frameworks of racism propose that racial/ethnic discrimination functions as chronic psychosocial stressors that are detrimental to health [8, 9]. This framework is supported by empirical evidence as racial discrimination is associated with a range of mental health problems including symptoms of depression and anxiety, substance use problems, and suicidality [10]. Further, research with longitudinal studies reported a prospective association between higher levels of racial/ethnic discrimination and mental health problems later in life [11]. Contemporary frameworks of racism suggest that racist-based incidents also manifest in online settings, and may be especially salient in the lives of young people [12].

According to the Cultural Theory and Model of Suicide [13], sociocultural contexts play a critical role in the development of suicide-related risk across the spectrum ranging from lower levels of risk (e.g., passive SI, or thoughts of suicide with no intent to die) to higher levels of risk (e.g., suicide death, or a fatal suicide attempt). Specifically, sociocultural context influences exposures to unique stressors that people from ethnoracially minoritized backgrounds encounter like racial/ethnic discrimination, acculturative stress, and other minority stressors. Sociocultural context also impacts the appraisal, response, and management of psychological distress. Lastly, sociocultural context shapes the attitudes toward and manifestations of suicidal thoughts and behaviors. Overall, the Cultural Theory and Model of Suicide proposes that suicide risk is not a universal process, but instead a culturally mediated one suggesting that a socioecological lens is critical to understanding suicide risk. Despite earlier calls for greater cultural considerations in suicide research [14], a lack of racial and ethnic diversity in suicide studies remains a problem [15]. As such, racism-related factors are seldom examined in the suicide literature. This limits the cultural responsiveness of existing suicide prevention strategies, particularly among ethnoracially minoritized college students.

Research suggests that experiences of racial and ethnic discrimination may confer suicide-related risk in part by increasing risk for psychological problems, including among ethnoracially minoritized college students in minority-serving institutions [16–19]. A systematic review of longitudinal studies further reported a significant prospective association between racial/ethnic discrimination and mental health problems including SI among adolescents and young adults [11]. Additionally, higher severity of SI among ethnoracially minoritized college students was positively associated with higher levels of racial discrimination as overt displays via institutional settings and as covert displays via daily microaggressions.

Drawing from the Cultural Theory and Model of Suicide [13] and the Biopsychosocial frameworks of racism-based stress [8, 9], we propose that the pervasive and distressing nature of experiences of racial/ethnic discrimination may exert a significant demand on the individual’s cognitive, emotional, biological, and psychological resources over time, which may, in turn, render the individual vulnerable to managing future distress engendering a suicidal crisis. Indeed, using cross-sectional data among ethnoracially minoritized college students in minority-serving institutions, researchers have identified mood and stress-related symptoms as potential explanatory factors in the link between racial/ethnic discrimination and suicide-related risk including hopelessness, symptoms of PTSD, depression, anxiety, dissociation, and stress sensitivity [16–19]. These studies, however, largely focused on offline racial/ethnic discrimination. Additionally, less is known about the potential role of sociocultural factors like ethnic identity and internalized racism that have been linked to psychological distress.

## Racial/Ethnic Discrimination and Alcohol Misuse

Experiences of racial/ethnic discrimination may also increase vulnerability for other health risk behaviors like alcohol misuse. The self-medication hypothesis posits that individuals may use alcohol, or other substances, to help cope with the distress and negative feelings associated with stressors [20]. In other words, the distress associated with racial/ethnic discrimination may exceed psychological resources and alcohol may be elicited to reduce tension and stress. Whereas substances like alcohol may offer a temporary alleviation of an aversive emotional state in the short-term, it may be overused as a coping strategy to develop into problematic use, or alcohol misuse, over time. Alcohol misuse may then yield more problems later in life like disruptions in relationships and work/school later in life, particularly with a chronic and pervasive psychosocial stressor like racial/ethnic discrimination.

Research shows that higher levels of racial/ethnic discrimination are associated with alcohol misuse among ethnoracially minoritized young adults. For instance, a meta-analysis investigating the link between racism-related experiences and alcohol misuse (e.g., alcohol consumption, binge/heavy drinking, at-risk drinking, alcohol use disorders, and negative drinking consequences) among Black American adults yielded 27 articles. The most common outcome variable identified was binge/heavy drinking, or 4/5 or more drinks in a short time frame [21]. The meta-analysis further shows that higher levels of racial/ethnic discrimination was positively associated with higher levels of alcohol consumption, heavy/binge drinking, at-risk drinking, and negative drinking consequences among Black adults supporting the self-medication theoretical framework. These associations, however, were not correlated with alcohol use disorder based on DSM IV criteria. Further, the association between negative drinking consequences and racial/ethnic discrimination was stronger at a younger age highlighting that young adults, including college students, may be at elevated risk.

Similar relations between racial/ethnic discrimination and alcohol misuse in other ethnoracially minoritized groups were reported in another systematic review investigating the association between racism-related experiences and a range of alcohol-related outcomes (e.g., drinking, binge episodes or other hazardous drinking patterns, negative consequences of drinking, and symptoms of dependence) among a racially and ethnically diverse sample of adolescents and adults resulting in 97 articles [22]. Review results indicate that racial/ethnic discrimination was the most frequently studied exposure variable in the literature, and that the majority of these studies shows a positive association between racial/ethnic discrimination and alcohol abuse and dependence.

Despite the review findings’ support for the link between racial/ethnic discrimination and alcohol misuse, no review included online racism experiences. Young people spend a significant amount of time interacting socially online, which creates another avenue wherein they are exposed to racism-related experiences [23], and thus, have increased vulnerability to cope with more hazardous drinking. For instance, Latine^2^ adolescents who experience higher levels of everyday racial/ethnic discrimination have the highest risk for past 30-day alcohol and other substance use during the transitional period from high school through emerging adulthood compared to those with lower, stable levels of racial/ethnic discrimination [24]. A similar trajectory between racial/ethnic discrimination and increased past 30-day alcohol use has been observed in a longitudinal Black emerging adult sample [25]. Interestingly, a longitudinal study found that Latine college students who experience racial/ethnic discrimination reported more alcohol misuse a year later, though alcohol misuse did not increase risk for later experiences of racial/ethnic discrimination [26], highlighting the deleterious mental health impact of discrimination and susceptibility for alcohol misuse following a distressing experience. Taken together, evidence suggests that young ethnoracially minoritized college students who experience racial/ethnic discrimination may be at increased risk for alcohol misuse.

## Online Racial Discrimination, Suicidal Ideation, and Alcohol Misuse

The existing research on racial/ethnic discrimination and health risk behaviors like SI and alcohol misuse has largely focused on in-person or offline exposures to racism. Thus, less is known about the role of online racial/ethnic discrimination (OR/ED) and risk for SI and alcohol misuse in ethnoracially minoritized college students. This is important as social media use has increased significantly among adolescents over the last decade, especially as Black and Latine adolescents spend more time engaging in social media use than their White counterparts [23].

A consequence of the increase in social media use among young people has been a new avenue for the proliferation of racism, considering the low oversight and high anonymity in online settings [12]. Like its offline counterpart, online racism is “a system of anti-people of color practices that privilege and maintain political, cultural, and economic power for Whites in digital space” [12]. The practices include creating algorithms that shape people’s experiences online (e.g., Black people disproportionately receiving ads for for-profit colleges and universities). They can also be reinforced interpersonally (e.g., racial harassment via verbal or text assaults on gaming, dating, chat, or social media platforms). It has a range of dimensions including online racial discrimination (e.g., denigrating or excluding individuals and groups on the basis of race) and traumatic racist events online (e.g., graphic images or videos of racism-based attacks) [12]. OR/ED can be direct where the exposed individual was the intended target of the racist-based incident or vicarious wherein indirect exposure results from someone from a shared background as the intended target.

OR/ED is prevalent, particularly among Black and Latine adolescents [27, 28], and increases over time [29]. It has been linked to mental health problems including symptoms of depression, anxiety, and traumatic stress in adolescents [27, 30–32] and young adults including college students [33–36], even when accounting for offline experiences of racial/ethnic discrimination [30]. In fact, an ecological momentary analysis tracking offline and online experiences of racial discrimination within a 2-week span among African American adolescents shows that ORD may be more prevalent than offline experiences of racial discrimination [28]. There is also early evidence suggesting that OR/ED may increase risk for alcohol misuse [37–39], and self-harming thoughts [40]. Together, these findings suggest that exposure to OR/ED may increase risk for SI and alcohol misuse in ethnoracially minoritized college students.

## Internalized Racism as a Risk Factor

Growing evidence shows the detrimental effects of racial/ethnic discrimination on mental health and psychological symptoms; however, less is known about sociocultural mechanisms underlying this association. One potential pathway through which experiences of racial/ethnic discrimination may increase vulnerability to health risk behaviors like SI and alcohol misuse is through internalized racism. As one of the myriad of psychological responses to the distress resulting from experiences of racial/ethnic discrimination to shape health-related outcomes [7], internalized racism may increase vulnerability to health risk behaviors by severing connections with values, beliefs, and practices that may be promotive and adaptive in the face of experiences of racism. Also referred to as “internalized self-hatred” or “internalized racial oppression,” internalized racism is the acceptance of negative societal beliefs and stereotypes about racially and ethnically minoritized groups. White supremacy ideologies are embedded in our social structures (i.e., institutions) via policies and practices that are largely maintained by individual actors via behavioral manifestations of racism in the form of discrimination (i.e., unjust treatment) as well as cognitive manifestations in the form of attitudes and biases (i.e., prejudices). As such, internalized racism results from frequent exposure to racism and also perpetuates racism by manifesting as discrimination and prejudice toward others and internally directed (i.e., self-hatred) [7]. Frequent exposure to racism during adolescence and into emerging adulthood is detrimental to mental health, as longitudinal research shows that cumulative exposures over time and early onset of racism exposure is associated with poor mental health outcomes later in life [11]. Further, emerging adulthood is a transitionary period characterized by fortified identity formation, greater independence, and engagement with new strata of society [41], which is especially salient in the lives of ethnoracially minoritized young people. Experiences of racial/ethnic discrimination may exert its harm on enthoracially minoritized college students in part by engendering internalized racism, which may increase their vulnerability to developing psychological distress.

Despite growing evidence linking racial/ethnic discrimination and risk for SI [42] as well as alcohol misuse [21], the extant literature has largely focused on interpersonal racism (e.g., discrimination). Thus, less is known about the role of other forms of racism like internalized racism. In recent years, however, research on the psychological effects of internalized racism has garnered significant attention. Findings from recent systematic reviews show that increases in experiences of racial/ethnic discrimination is associated with increases in internalized racism [43–46], and that internalized racism negatively impacts mental health [44, 45]. For instance, in a longitudinal study of African American college students attending a Predominantly White Institution, researchers reported that higher levels of racial discrimination prospectively predicted higher levels of internalized racism a year later [46]. Another study reported that higher levels of internalized racism was prospectively associated with psychological distress (i.e., depression and anxiety symptoms) in a longitudinal study of African American young adults [47]. Additionally, another study found a significant association between internalized racism and polysubstance use including alcohol use, even after accounting for institutional and interpersonal racism-related experiences, among Black young adults [48].

There is also empirical evidence suggesting that internalized racism may influence the association between racial/ethnic discrimination and SI in adults [49, 50] including among Asian American young adult women [51]. Among Black/African American women, internalized racism was positively associated with increased alcohol consumption [52] and similar findings were reported among Asian American men [53]. In another study documenting daily changes in racial discrimination, internalized racism, and sleep for about a week among a group of African American college students, researchers found that those reporting higher levels subsequently experienced more sleep problems, but only among those reporting higher levels of internalized racism [54]. These studies, however, examined internalized racism as a moderator in the link between discrimination and SI/substance use suggesting that discrimination may increase risk for SI/substance use link among those with internalized racism. This approach presumes an independent relation between racial/ethnic discrimination and internalized racism. Frameworks of internalized racism, however, propose that internalized racism may result from accumulating experiences of racism or discrimination over time. Considering that experiences of racism are pervasive and salient in the lives of ethnoracially minoritized youth [11, 27–29], it is possible that internalized racism may function as a potential pathway through which discrimination may confer risk for health behaviors. Thus, perhaps OR/ED may increase vulnerability to health risk behaviors through increases in internalized racism among college students. Additionally, other sociocultural factors like ethnic identity may influence which individuals experiencing racial discrimination go on to exhibit internalized racism. However, to our knowledge, no study to date has examined internalized racism as a potential explanatory factor in the association between OR/ED and SI/alcohol misuse.

## Ethnic Identity as a Protective Factor

Racial/ethnic discrimination experiences, both online and offline, are prevalent and pervasive, nevertheless, not all individuals will internalize these racist ideologies. Similarly, the majority of individuals exposed to racism will not develop mental health problems. This may be explained, in part, by adaptations that people from ethnoracially minoritized groups develop over time to ward off the harmful effects of racism. One of these adaptations may be the development of a strong racial or ethnic identity, or an individual’s beliefs, attitudes, and sense of belonging and attachment to their ascribed racial and/or ethnic group [55]. The Cross Ethnic-Racial Identity Model acknowledges the dimensionality of ethnoracial identity and includes a stage-based, developmental approach starting at self-rejection and ending at the evolved stage of self-acceptance. Such changes may be facilitated by aspects of ethnic identity involving the exploration of one’s ethnic group affiliation characterized by engaging in cultural practices and actively seeking out knowledge about their ethnic group [56]. Following the exploration phase, one may reject their ethnic group membership altogether or develop a sense of attachment and belonging that ranges from weak to strong, which is referred to as ethnic identity commitment. A strong ethnic identity may promote adaptive coping skills in the face of racial/ethnic discrimination by providing social and emotional support from adults and peers with a shared background, encouraging cognitive appraisals like external rather than internal attributions to racist-based incidents, promoting self-esteem and connection, and the meaning that they ascribe to their group membership [57].

Growing research shows that increased attachment and sense of belonging to one’s racial/ethnic group may function as a protective factor against the harmful effects of racial/ethnic discrimination [58], including OR/ED [30, 59]. Indeed, prior research shows that increases in levels of ethnic identity attenuated the association between offline racial/ethnic discrimination and SI [19]. Similarly, experiences of racial/ethnic discrimination were less strongly related to alcohol consumption among Black/African American college students who reported a strong or positive racial/ethnic identity [60]. Further, different dimensions of ethnic identity may differentially associate with health outcomes. Specifically, there is empirical evidence suggesting an association between commitment (i.e., a strong sense of attachment to one’s ethnic group) and positive health outcomes, whereas exploration (i.e., engaging in cultural behaviors and practices connected to one’s ethnic group) is associated with negative health outcomes, though findings show that the effects of ethnic identity are influenced by race/ethnicity, age, and other contextual factors [58]. Thus, clarifying the dimensions of ethnic identity that may render an individual more vulnerable or resilient to experiences of racial/ethnic discrimination will inform potential pathways wherein discrimination may influence risk for SI or alcohol misuse.

## The Present Study

Using a cross-sectional, survey-based study of ethnoracially minoritized college students from a Hispanic-Serving Institution in a metropolitan area in Northeastern U.S., the present study is the first to date to examine the direct and indirect associations between OR/ED and SI and alcohol misuse through internalized racism, and whether dimensions of ethnic identity, exploration or commitment, differentially moderate these associations. We hypothesized that greater levels in frequency of OR/ED, individual and vicarious, would be positively associated with greater severity of SI, greater alcohol misuse assessed as frequency of alcohol use, and internalized racism. We also examined internalized racism as a potential pathway in the relation between OR/ED and SI and alcohol misuse. Thus, we further hypothesized that these associations would be explained, in part, by higher levels of internalized racism. Lastly, we examined ethnic identity as a moderator in the association between OR/ED and internalized racism, SI, and alcohol misuse. We hypothesized that increases in ethnic identity commitment will attenuate, whereas exploration would exacerbate the associations between OR/ED and internalized racism on SI and alcohol misuse.

## Method

### Participants

Participants were drawn from a larger study examining sociocultural factors and mental health symptoms among undergraduate students from a public, Hispanic-Serving Institution in a Northeastern metropolitan area in the U.S. The initial sample consisted of 838 students who completed an online survey [61]. Eleven participants were removed from analyses due to self-reported age being above 30 years. We limited the sample to those below age 30 years, as evidence suggests that emerging adulthood (i.e., ages 18–30 years) coincides with the peak onset of most psychological disorders [62]. This left us with a sample of 827 undergraduate students (80.9% female), aged 18 to 30 years old (*M* = 19.61, *SD* = 2.03). The original sample was ethnoracially diverse with 37.8% self-identifying as Hispanic/Latino/a/x/e, 38.2% non-Latine White, 12.9% as non-Latine Black, 7.3% as non-Latine Asian, 0.4% Native Hawaiian/Pacific Islander, 0.2% as Native American/American Indian, 2.1% Multiracial, and 0.4% Other.

For the current study, the analytic sample consisted of participants who self-identified as an ethnoracially minoritized individual (*n* = 513). Of these, 78.6% were female with a mean age of 19.62 (*SD* = 2.08). About 60% self-identified as Latine, 20.7% non-Latine Black, 12.3% non-Latine Asian American or Pacific Islander (AAPI), 3.9% Multiracial, 1.8% Middle Eastern/North African (MENA), 0.2% Native American/American Indian, and 0.2% identified as other ethnoracially minoritized background. Freshmen students made up 54% of the sample. The majority of participants (83.2%) indicated being born in the U.S. Most students (77.0%) reported receiving federal or state financial assistance for their college education.

### Procedure

Participants were recruited through an online subject pool to earn course credits and through flyers posted on campus. The survey was administered online using Qualtrics during the Spring and Fall 2019 semesters, prior to the COVID-19 pandemic. Participants who completed the survey either received one credit or a $5 gift card. Informed consent was obtained electronically. The Institutional Review Board at the participating institution approved these study procedures.

#### Suicidal Ideation

The Beck Scale for Suicide Ideation (BSS) [63] is a 19-item scale assessing current suicidal ideation. Participants rate items on a three-point scale describing how they feel during the past week (e.g., “I have a moderate to strong wish to live; I have a weak wish to live; I have no wish to live”). A total score was computed to assess the severity of SI. The BSS has been used with racially and ethnically diverse samples of college students and demonstrated excellent reliability [19]. The scale scores demonstrated good internal reliability in the present sample ($\alpha$ = 0.84).

#### Alcohol Misuse

The Alcohol Use Disorders Identification Test (AUDIT) [64] is a 10-item survey that assesses alcohol consumption, drinking behavior or dependence, and alcohol-related problems and consequences in the past year. Participants were asked the frequency in which they consume alcohol (e.g., “How often do you have a drink containing alcohol?”), with response options ranging from never (0) to four or more times a week (4). Scale scores were computed as the sum of all the items. The AUDIT is a reliable and valid instrument for detecting high-risk alcohol use among college students [65, 66].

#### Online Racial and Ethnic Discrimination

The Online Victimization Scale (OVS) [67] is a 21-item measure that examines online victimization in the past year and consists of 4 subscales: General, Sexual, Individual Online Racial Discrimination, and Vicarious Online Racial discrimination. Participants rate items using the following six response options: 1 (never happened), 2 (happened once), 3 (a few times a year), 4 (a few times a month), 5 (a few times a week), and 6 (occurs daily). We used the Individual Racial Discrimination and Vicarious Online Racial Discrimination subscales. The Individual Online Racial Discrimination subscale consists of 4-items that assess online discrimination aimed directly at the respondent (e.g., “People have said mean or rude things about me because of my race or ethnic group online”). Four items comprise the Vicarious Online Racial Discrimination subscale, which examines experiences of online discrimination directed at other people that the respondent has observed (e.g., “I have witnessed people saying mean or rude things about another person’s ethnic group online.”). Subscale scores were computed as the sum of all items. Previous studies have confirmed the factor structure of the OVS and has indicated strong validity and internal consistency [27, 29]. The OVS has recently been used in research with ethnoracially minoritized college students and the individual and vicarious discrimination subscales have demonstrated good reliability [35]. In the present sample, scale scores were good for the Individual and Vicarious Online Racial Discrimination subscales ($\alpha$ = 0.73) and ($\alpha$ = 0.75), respectively.

#### Internalized Racism

The Cross Ethnic-Racial Identity Scale (CERIS) [68] is a 29-item scale that evaluates racial/ethnic identity attitudes. Participants rate items using a 7-point Likert-type scale from 1 (strongly disagree) to 7 (strongly agree). We used the 4-item Self-Hatred subscale to assess internalized racism. This subscale measures the extent to which individuals dislike the ethnic-racial group to which they belong (e.g., “When I look in the mirror, sometimes I do not feel good about the ethnic/racial group I belong to”). The Self-Hatred subscale demonstrates adequate reliability and validity in a non-clinical sample [68] and has been used in previous research examining internalized racism among Black college students [69]. Subscale scores were computed as the sum of all items. The subscale scores for this sample were excellent ($\alpha$ = 0.92).

#### Ethnic Identity

The Multigroup Ethnic Identity Measure-Revised (MEIM-R) [70] was used to assess participants’ identification with their ethnic group, which demonstrates strong psychometric properties comparable to the original, longer version of the scale. This 6-item measure consists of two subscales: exploration and commitment. Three of these items assess exploration, which refers to an individual’s efforts to learn more about their ethnic group and to participate in the cultural practices of their ethnic group (e.g., “I have spent time trying to find out more about my ethnic group, such as its history, traditions, and customs.”). The remaining three items assess commitment, which reflects positive affirmation of one’s ethnic group and a sense of commitment to the group (e.g., “I have a strong sense of belonging to my own ethnic group.”). Participants rate items using a 5-point Likert-type scale ranging from 1 (strongly disagree) to 5 (strongly agree). Subscale scores were computed as the sum of all items. In the present sample, the scale scores were good for the exploration and commitment subscales ($\alpha$ = 0.87) and ($\alpha$ = 0.92), respectively.

### Data Analysis

Analyses were conducted using IBM SPSS Version 27. Missing data were excluded from analyses using listwise deletion. Preliminary checks identified no multivariate outliers or any violations of the assumptions of normality or multicollinearity. Independent samples *t*-tests and chi-square tests were used to examine differences between participants with missing data and those with complete data. Descriptive statistics, including means and standard deviations for continuous variables and frequencies for categorical variables, were computed. Pearson’s correlation was used to examine bivariate associations between all variables in the study. We then conducted independent sample *t*-tests to examine differences in the main study variables by gender and one-way analysis of variance (ANOVA) to examine differences by race/ethnicity. To test the study aims, we computed separate models for each outcome: SI and alcohol use. First, to test the direct and indirect effects of individual online racial/ethnic discrimination and SI or alcohol misuse through internalized racism, we used model 4 from the statistical macro for SPSS PROCESS version 4.2 [71]. In this model, the indirect effect of individual OR/ED on SI and alcohol misuse via internalized racism and its 95% confidence interval (CI) was estimated using 10,000 bootstrapped samples. For this procedure, a 95% CI that did not include zero was considered statistically significant. A similar model was computed to test whether internalized racism had a direct and indirect effect on the relation between vicarious OR/ED and SI and alcohol misuse. Analyses were adjusted for sex and race/ethnicity.

Next, model 7 of Hayes’ PROCESS 4.2 SPSS macro was used to test the full hypothesized model with ethnic identity commitment and exploration as separate moderators of the path from OR/ED to internalized racism [71]. In cases of significant moderation, the direct and indirect effects of OR/ED on internalized racism at the 16th, 50th, and 84th percentiles of ethnic identity were investigated using the Johnson-Neyman technique to determine the nature of the interactions. All analyses adjusted for sex and race/ethnicity.

## Results

### Descriptive and Bivariate Analyses

Of the 513 participants who self-identified as an ethnoracially minoritized individual, 494 (96.3%) provided complete data on the variables of interest. No significant differences on demographic variables or mental health symptom measures emerged between the analytic sample and those excluded due to missing data. Descriptive statistics for the analytic sample are listed in Table 1. Approximately 16.4% (*n* = 81) of the sample endorsed a recent episode of SI on the BSS (score > 0 on either item 4 and/or item 5), with the mean BSS score being 1.62 (*SD* = 3.67; range = 0–28). The mean score for the AUDIT was 1.91 (*SD* = 2.76; range = 0–14). No significant sex differences in SI (*t* = 1.06, *p* = 0.289) or alcohol misuse (*t* = 1.93, *p* = 0.056) emerged. Additionally, there were no significant racial/ethnic differences in SI (I(3, 490) = 1.01, *p* = 0.389) or alcohol misuse (*F*(3, 490) = 1.26, *p* = 0.287). Although no significant sex differences emerged regarding individual OR/ED (*t* = 1.06, *p* = 0.289), males (*M* = 5.03, *SD* = 3.74) reported experiencing significantly more vicarious OR/ED than females (*M* = 4.09, *SD* = 3.37; *t* = 2.47, *p* = 0.014). The experience of individual OR/ED and vicarious OR/ED also varied by race/ethnicity (*F*(3, 490) = 14.77, *p* < 0.001; *F*(3, 490) = 2.99, *p* = 0.031, respectively). Post hoc Bonferroni analyses indicated that Black and Other students were significantly more likely to report individual OR/ED than Asian and Latine students. Additional post hoc Bonferroni analyses did not reveal significant differences in vicarious OR/ED by race/ethnicity. Finally, females (*M* = 11.18, *SD* = 3.19) reported higher levels of ethnic identity commitment than males (*M* = 10.42, *SD* = 3.30; *t* =  − 2.16, *p* = 0.032). No other sex differences in the variables examined were observed.

**Table 1:** Sample characteristics

Variable	*n* (%)
Sex (assigned at birth)	
Female	388 (78.5)
Male	105 (21.3)
Prefer not to disclose	1 (0.2)
Race/ethnicity	
Non-Latinx Black	98 (19.8)
Non-Latinx Asian American	59 (11.9)
Hispanic/Latino/a/e/x	305 (61.7)
Multiracial	19 (3.8)
Native Hawaiian/Pacific Islander	2 (0.4)
Native American/American Indian	1 (0.2)
Middle Eastern/North African	9 (1.8)
Other	1 (0.2)
Year in school	
Freshman	268 (54.3)
Sophomore	106 (21.5)
Junior	86 (17.4)
Senior	34 (6.9)
Sexual orientation	
Straight/heterosexual	405 (82)
Gay	12 (2.4)
Lesbian	11 (2.2)
Bisexual	48 (9.7)
Prefer to self-identify	9 (1.8)
Prefer not to disclose	9 (1.8)
Receiving financial aid	
Yes	379 (76.7)
No	91 (18.4)
Prefer not to disclose	24 (4.9)

Findings from the Pearson correlation analyses are displayed in Table 2. Individual and vicarious OR/ED were positively associated with alcohol misuse, SI, internalized racism and ethnic identity exploration though not commitment. Only vicarious OR/ED was significantly positively associated with ethnic identity commitment. Internalized racism was positively associated with SI, but not alcohol misuse. Ethnic identity exploration and commitment were both negatively associated with SI and alcohol misuse.

**Table 2:** Bivariate correlations among main study variables

	1	2	3	4	5	6	7
1. Individual ORD	-						
2. Vicarious ORD	.526**	-					
3. Internalized racism	.233**	.186**	-				
4. MEIM-E	.121**	.221**	− .021	-			
5. MEIM-C	.064	.171**	− .176**	.766**	-		
6. Suicidal ideation	.156**	.139**	.197**	− .119**	− .211**	-	
7. Alcohol use	.133**	.142**	.076**	− .128**	− .161**	.290**	-
*M*(*SD*)							
Females	1.91 (2.65)	4.09 (3.37)	7.28 (4.70)	10.36 (3.20)	11.18 (3.19)	1.52 (3.63)	1.77 (2.57)
Males	2.12 (2.86)	5.03 (3.74)	7.19 (4.98)	9.79 (3.32)	10.42 (3.30)	1.95 (3.82)	2.45 (3.34)
*t*	0.722	2.47*	− 0.178	− 1.60	− 2.16	1.06	1.93*

*p < .05; **p < .001

### Simple Mediation Analyses

#### Individual Online Racial/Ethnic Discrimination

The results of the simple mediation analyses examining the indirect effect of individual OR/ED on SI and alcohol misuse adjusted by sex and race/ethnicity are presented in Table 3. The findings (model 1) indicated that individual OR/ED positively predicted internalized racism (*B* = 0.40, *p* < 0.001). Thus, students who experienced more individual OR/ED reported greater levels of internalized racism. Moreover, the results (see models 2 and 3) indicated that internalized racism positively predicted SI (*B* = 0.18, *p* < 0.001) but not alcohol misuse (*B* = 0.03, *p* = 0.25). Students who had higher levels of internalized racism were more likely to report SI, but not greater alcohol misuse. Additionally, individual OR/ED directly predicted SI (*B* = 0.28, *p* < 0.001) and alcohol misuse (*B* = 0.13, *p* = 0.004). Bootstrapping analyses indicated that the indirect effect of individual OR/ED on SI through internalized racism was significant (indirect effect = 0.07, *SE* = 0.03, 95% CI = [0.03–0.13]). These results suggest that students who experienced more individual OR/ED were prone to higher levels of internalized racism, which placed students at higher risk of SI. The indirect effect of individual OR/ED on alcohol misuse through internalized racism was not significant (indirect effect = 0.01, *SE* = 0.01, 95% CI = [− 0.01–0.04]).

**Table 3:** Indirect effect of individual online racial discrimination on suicidal ideation and alcohol use via internalized racism

Predictors	Model 1 (internalized racism)		Model 2 (suicidal ideation)		Model 3 (alcohol use)	
	*B* (SE)	*t*	*B* (SE)	*t*	*B* (SE)	*t*
Individual ORD	.40 (.08)	5.08**	.28 (.06)	4.59**	.13 (.05)	2.64*
Internalized racism			.18 (.03)	5.34**	.03 (.03)	1.15
*R*^*2*^	.06		.12		.04	

All models adjust for sex and race/ethnicity; *p < .01; **p < .001

#### Vicarious Online Racial/Ethnic Discrimination

Table 4 presents the findings of the simple mediation analyses examining the indirect effect of vicarious OR/ED on SI and alcohol misuse adjusted by sex and race/ethnicity. The findings (model 1) indicated that vicarious OR/ED positively predicted internalized racism (*B* = 0.26, *p* < 0.001). Students who experienced more vicarious OR/ED reported greater levels of internalized racism. The results (see models 2 and 3) also indicated that internalized racism positively predicted SI (*B* = 0.20, *p* = 0.003) but not alcohol misuse (*B* = 0.03, *p* = 0.201). Vicarious OR/ED also directly predicted SI (*B* = 0.14, *p* = 0.047) and alcohol misuse (*B* = 0.10, *p* = 0.005). Bootstrapping analyses indicated that the indirect effect of vicarious OR/ED on SI through internalized racism was significant (indirect effect = 0.05, *SE* = 0.02, 95% CI = [0.02–0.09]). These results suggest that students who experienced more vicarious OR/ED were prone to higher levels of internalized racism, which placed students at higher risk of SI. The indirect effect of vicarious OR/ED on alcohol misuse through internalized racism was not significant (indirect effect = 0.01, *SE* = 0.01, 95% CI = [− 0.01–0.03]).

**Table 4:** Indirect effect of vicarious online racial discrimination on suicidal ideation and alcohol use via internalized racism

Predictors	Model 1 (internalized racism)		Model 2 (suicidal ideation)		Model 3 (alcohol use)	
	*B* (SE)	*t*	*B* (SE)	*t*	*B* (SE)	*t*
Vicarious ORD	.26 (.06)	4.19**	.14 (.05)	2.97*	.10 (.04)	2.80*
Internalized racism			.20 (.03)	5.79**	.03 (.03)	1.28
*R*^2^	.04		.10		.03	

All models adjust for sex and race/ethnicity; *p < .01; **p < .001

### Moderated Mediation Models

#### Individual Online Racial/Ethnic Discrimination

The results of the full moderated mediation model examining if ethnic identity exploration moderates the indirect effect of individual OR/ED on SI and alcohol misuse via internalized racism are summarized in Supplemental Table 1. The index of moderated mediation was not statistically significant for SI (index =  − 0.003, *SE* = 0.01, 95% CI [− 0.02–0.01]) or alcohol misuse (index =  − 0.001, *SE* = 0.001, 95% CI [− 0.004–0.001]). The model examining whether ethnic identity commitment moderates the indirect effect of individual OR/ED on SI and alcohol misuse is presented in Supplemental Table 2. The index of moderated mediation for exploration was also not statistically significant for SI (index =  − 0.01, *SE* = 0.01, 95% CI [− 0.03–0.004]) or alcohol misuse (index =  − 0.001, *SE* = 0.003, 95% CI [− 0.009–0.002]).

#### Vicarious Online Racial/Ethnic Discrimination

**Fig. 1:**
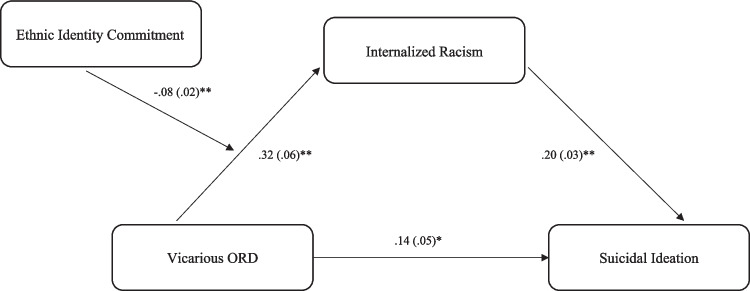
Final moderated mediation model evaluating whether ethnic identity commitment moderates the indirect effect of vicarious online racial discrimination on SI

Supplemental Table 3 summarizes the results of the full moderated mediation models examining if ethnic identity exploration moderates the indirect effect of vicarious OR/ED on SI and alcohol misuse via internalized racism. The index of moderated mediation for exploration was not significant for SI (index =  − 0.006, *SE* = 0.004, 95% CI [− 0.015–0.001]) or alcohol misuse (index =  − 0.001, *SE* = 0.001, 95% CI [− 0.005–0.001]). Supplemental Table 4 presents the results for the model evaluating whether ethnic identity commitment moderates the indirect effect of vicarious OR/ED on SI and alcohol misuse. The index of moderated mediation was statistically significant for SI (index =  − 0.015, *SE* = 0.006, 95% CI [− 0.028 to − 0.007]). At low and average levels of ethnic identity commitment, there was an indirect effect of vicarious OR/ED on SI via internalized racism. However, at high levels of commitment, there was no indirect effect present (see Fig. 1 for visual presentation of the overall moderated mediation). The index of moderated mediation was not statistically significant for alcohol misuse (index =  − 0.003, *SE* = 0.003, 95% CI [− 0.008–0.002]).

## Discussion

In the past decade, there has been a disproportionate rise in mental health problems including suicidal ideation (SI) among ethnoracially minoritized college students [3]. Alcohol misuse has also increased during this same time period among this population [4, 5]. Recent research shows that online racism is a growing problem among young people from ethnoracially minoritized backgrounds [27–29], and may help explain the elevated rates of mental health problems in college students, particularly from ethnoracially minoritized backgrounds. This study aimed to examine the direct and indirect associations between online OR/ED and health risk behaviors (i.e., SI and alcohol misuse) among ethnoracially minoritized college students. We also examined whether internalized racism helps explain these associations, and whether ethnic identity exploration or commitment moderated the indirect association between OR/ED on SI and alcohol misuse via internalized racism. Our first hypothesis was supported as we found that both individual and vicarious OR/ED were directly associated with higher levels in SI, alcohol misuse, and internalized racism. Our second hypothesis was also supported as we found that an indirect association between individual OR/ED and SI was detected via higher levels of internalized racism. Similarly, there was an indirect association between vicarious OR/ED and SI through internalized racism. Our third hypothesis was not supported, however, as these indirect associations were not observed with alcohol misuse. Our final hypothesis was partially supported as ethnic identity commitment moderated the indirect association of vicarious, though not individual, OR/ED on SI via internalized racism such that the pathway of vicarious OR/ED to SI via internalized racism was detected among those with low and moderate, but not high, levels of ethnic identity commitment. No other moderation effects of ethnic identity (i.e., commitment or exploration) emerged as significant in the other hypothesized associations.

Our study findings broaden our understanding of the association between online racism and health risk behaviors by clarifying the potential role of internalized racism and ethnic identity. These findings are consistent with an earlier study reporting a direct association between online racial discrimination and self-harming thoughts among young adults [36], and extends it to a recent SI episode as previously reported among Black and Latine adolescents [72, 73]. We expand on this prior research by demonstrating that internalized racism may help explain, in part, the association between OR/ED, individual and vicarious exposures, and current SI. Therefore, OR/ED may confer risk for thinking about suicide among ethnoracially minoritized college students to the extent that it increases their internalization of racist-based beliefs. This finding further expands on the existing literature demonstrating a link between racial/ethnic discrimination, albeit offline, and SI among ethnoracially minoritized college students [16–19, 42]. The association between individual OR/ED and SI also corroborates prior research showing a well-documented link between online racism and mental health problems [27, 30, 33] including suicidal ideation [72, 73]. The literature on vicarious OR/ED, however, has been mixed. For instance, in one study, researchers reported vicarious OR/ED was associated with lower levels of depression symptoms among Latine adolescents [30]. Meanwhile, another study with Latine young adults reported a positive association between OR/ED and depression symptoms [33]. Additionally, a recent study among Latine adolescents reported no association between vicarious OR/ED and SI [72]. Our study further supports a significant positive link between vicarious OR/ED and SI. Thus, whereas growing evidence shows a link between direct exposures to OR/ED and SI, further research is warranted to help explain the discrepant findings regarding vicarious OR/ED. Perhaps age-related differences in the prevalence and impact of vicarious OR/ED may help explain this discrepancy as exposures have been shown to increase with age [29].

Our findings are also consistent with prior research demonstrating that internalized racism may influence the association between racial discrimination and SI as reported in a sample of Asian American young adult women [51], though this study examined internalized racism as a potential moderator of the association and examined offline racial discrimination. Specifically, Keum and colleagues [51] examined three distinct dimensions of internalized racism (i.e., self-hatred, appearance bias, weakness stereotype) as a potential moderator in the discrimination-SI link, and only found evidence for self-hatred. This approach, however, presumes an independent relation between racial discrimination and internalized racism, though frameworks propose that internalized racism is likely the result of frequent exposure to racism [7, 68]. Indeed, prior research shows that higher levels of racial/ethnic discrimination experiences, albeit offline, is prospectively associated with higher levels of internalized racism a year later [46], and that higher levels of internalized racism is prospectively associated with higher levels of psychological distress and mental health problems [44, 45, 47]. Our study is the first to date to demonstrate the association between internalized racism and SI across both individual and vicarious exposures of OR/ED. Further longitudinal research is warranted to better understand the temporality in the role of internalized racism in the association between OR/ED and SI. Overall, our findings suggest that direct and indirect exposures to OR/ED may help explain the elevated risk for SI among ethnoracially minoritized college students to the extent that it may increase internalized racism, and thus, OR/ED may be a relevant risk factor for SI among this population.

We also found that ethnic identity may mitigate the harmful effects of OR/ED, though this effect was observed for vicarious but not individual OR/ED. Specifically, we observed that higher levels of ethnic identity commitment may buffer the harmful effects of vicarious, but not individual, experiences of OR/ED on SI through internalized racism. This is consistent with prior research showing the protective qualities of ethnic identity on mental health and self-esteem when exposed to OR/ED [33, 59] and offline racial discrimination [19]. This research, however, did not examine the differential impact of ethnic identity subdomains. Our findings broaden the existing literature by demonstrating that ethnic identity commitment, though not exploration, may yield protective effects to help mitigate the harmful impact of vicarious OR/ED on SI risk among ethnoracially minoritized college students. Perhaps having a strong sense of connection and attachment to one’s ethnic identity may prepare these college students with the necessary coping skills to prevent the internalization of racism following vicarious exposures to online racism where someone else from a similar racial or ethnic background is the intended target of the attack [57]. The moderating effect of ethnic identity on the association between vicarious OR/ED and mental health has been reported by previous research. For instance, one study reported that greater levels of ethnic identity weakened the association between vicarious, though not individual, OR/ED and anxiety symptoms among Latine young adults [33].

A surprising finding in our study was that ethnic identity commitment did not moderate the association between individual OR/ED and internalized racism. This is consistent with prior research showing that higher levels of racial identity in the form of private regard (i.e., one’s personal beliefs about their racial group) weakened the association between indirect, though not direct, exposures to online racism and posttraumatic stress symptoms among a nationally representative sample of Black and Latine adolescents [74]. One possible explanation may be that individuals with higher ethnic identity may be more attuned to perceiving experiences of racism, as some dimensions of ethnic identity are associated with negative mental health outcomes [58]. Considering that ethnic identity is a multidimensional construct [55–58], it is possible that the various dimensions of ethnic identity may differentially influence the association between OR/ED, internalized racism, and SI, and thus, dimensions not examined in this study may be more relevant to understanding the relation between individual OR/ED and internalized racism. It is also possible that the negative impact of direct exposures (i.e., individual) of OR/ED is such that even a strong ethnic identity is not enough to mitigate its harm, as OR/ED was associated with internalized racism at low, moderate, and high levels of ethnic identity commitment. Perhaps the psychological distress that results from direct OR/ED may be such that a more proactive and deliberate intervention beyond developing a positive attachment with their racial or ethnic group is warranted. An intervention that directly targets online racism may be more protective to disrupt the internalization of racism following direct OR/ED exposures such as Critical Race Digital Literacy Skills [75]. Introduced by Tynes and colleagues [75], Critical Race Digital Literacy Skills aims to prepare students with the necessary critical and analytical skills to consume and interpret race-based content online to avoid internalization. Ultimately, the goal of this curriculum is to empower students to engage with race-based content in ways that mitigate the harm and dangers of racism that manifest in technological and digital settings.

Consistent with prior research on alcohol use [37–39], we observed a direct association between OR/ED, both vicarious and individual, with alcohol misuse. Unlike with SI, however, we did not observe an indirect association between OR/ED alcohol misuse via internalized racism. Thus, online racism may confer risk for alcohol misuse among ethnoracially minoritized college students, but pathways explaining this association may not involve internalized racism. This is in contrast to prior research supporting internalized racism as a potential pathway from racial discrimination, albeit offline, and alcohol misuse [52, 53]. Instead, prior research suggests that psychological distress [37, 38], and depression and anxiety symptoms [39] may be more relevant to understanding the link between OR/ED and alcohol misuse. Another possible explanation may be the cultural context of the college environment, as one study found that Latine students attending a Hispanic-Serving Institution, compared to a non-Hispanic-Serving Institution, reported significantly less alcohol misuse [76]. Relatedly, our findings align with the “cross-over effect” where substance use and related problems are low in young adulthood among ethnoracially minoritized groups [77], and provides an opportunity to identify those at elevated risk for future alcohol-related problems.

### Limitations, Future Directions, and Clinical Implications

The present findings should be interpreted with several limitations in mind. First, the cross-sectional nature of the design limits our ability to draw causal inferences and the temporal order of the variables cannot be established. Thus, it is possible that factors underlying vulnerability to SI and alcohol misuse may similarly influence internalization of racist beliefs and development of ethnic identity. Nevertheless, we adhere to the recommended guidelines provided by Hayes [71] and Kraemer and colleagues [78] when using cross-sectional data to examine a developmental psychiatric process including clarity of terminology, valid, and reliable tools, and the type and timeframe of the constructs examined are conceptually supported. Specifically, OR/ED was assessed in the past year, internalized racism and ethnic identity was assessed as a trait-level factor, and our outcome variables (i.e., SI) was assessed as severity in the past week. Further, previous research employing longitudinal designs have demonstrated a prospective relation between offline racial/ethnic discrimination, internalized racism, SI, and alcohol misuse supporting our conceptual model of the temporal relation between the variables. The present findings serve as preliminary evidence in identifying potential risk (i.e., internalized racism) and protective (i.e., ethnic identity) factors in the association between online racial/ethnic discrimination and health risk behaviors (i.e., SI, alcohol misuse) among ethnoracially minoritized college students in minority-serving institutions. Future research examining the indirect association between online racial discrimination and health risk behaviors including SI and alcohol misuse with longitudinal data are warranted.

Another limitation of the study is that the findings may not generalize to the larger young adult or college student population, as the sample was predominantly female, Latine, and U.S.-born recruited from one university in Northeastern U.S. Further research is warranted to examine group differences across sex, sexual orientation, gender identity, race/ethnicity, and immigration status. Another limitation of the study is that offline racial discrimination experiences were not accounted for, thus, further research is warranted about the unique impact of online racial discrimination on health risk behaviors like SI and alcohol misuse. Lastly, the data was collected exclusively via self-report, which is subject to recall and social desirability bias.

Despite these limitations, the study has numerous strengths and offers important clinical implications. First, by focusing on OR/ED, we examine a growing problem that is common in the lives of many college students, particularly those from ethnoracially minoritized backgrounds. Second, the findings offer preliminary evidence for potential risk and protective factors that could help refine identification of students who are most vulnerable to thinking about suicide or engaging in alcohol use. Third, attending to racism-related experiences including OR/ED, internalized racism, and ethnic identity could help improve the cultural responsiveness of assessment, diagnosis, and treatment that is tailored to addressing health risk behaviors like SI and alcohol misuse in college student populations at minority-serving institutions.

The study findings can inform suicide and alcohol misuse prevention strategies to promote mental well-being on college campuses. For instance, it can inform college counselors and other mental health professionals who work closely with ethnoracially minoritized college students who are struggling with suicidal thoughts or alcohol misuse. Specifically, screening for online racism experiences may help identify students who are vulnerable to mental health problems, and promoting ethnic identity may be one way to help mitigate this harm. Another example is to support student affinity groups, clubs, and other organizations that foster connection and sense of belonging that affirms a student’s ethnic identity. College administrators and personnel can implement pedagogical approaches to navigating online settings attuned to racist-based content such as the Critical Race Digital Literacy Skills curriculum developed by Tynes and colleagues [75]. Lastly, companies creating and maintaining these technological structures and online platforms bear great responsibility for the harmful exposures like online racism in these digital settings. To support the well-being of our college students, these platforms must create safeguards to better screen out online racism and other harmful exposures.

Besides ethnic identity, researchers have identified other factors that may help mitigate the harmful effects of OR/ED, which warrant further investigation in relation to SI and alcohol misuse. Such factors include racial/ethnic socialization (i.e., messages exchanged between caregivers and their offspring about race and racism) [79], critical race digital literacy, activism [80], and civic engagement [81].

## Conclusion

Ethnoracially minoritized college students may be especially vulnerable to mental health problems including SI and alcohol misuse. Emerging research shows that OR/ED experiences may negatively impact the mental health of young people, but potential pathways underlying this association remain understudied. The present study found a direct association between OR/ED with SI, alcohol misuse, and internalized racism. We also found an indirect association between OR/ED and SI, though not alcohol misuse, via internalized racism. Ethnic identity commitment attenuated this indirect association, but only with vicarious, though not individual, OR/ED. Our findings further highlight the critical role of OR/ED among ethnoracially minoritized college students and identify potential pathways through which said experiences may confer risk for SI.

## Supplementary Information

Below is the link to the electronic supplementary material.

Supplementary file1 (DOCX 17 KB)

## Data Availability

Data available upon request.

## Footnotes

1 We use the term ethnoracially to reflect individuals who identify from a racially and ethnically minoritized background, e.g., Black/African American, Hispanic/Latina/o/x/e, Asian American, Pacific Islander, Native American/American Indian/Native Hawaiian/Alaskan, Middle Eastern/North African. We acknowledge that race and ethnicity are two distinct social constructs that are often conflated and used interchangeably by laypeople in real-world settings, and that experiences of discrimination are not easily distinguishable between race and ethnicity.

2 We use the term Latine as a gender inclusive term to refer to people from Latin American and Caribbean background. Latine can be pronounced and conjugated in Spanish and other Latin languages, differently from the alternative Latinx, mostly used in the U.S. We acknowledge as a main limitation of these terms that at this point in time they are not being widely used by the majority of the members of the communities that they aim to represent.
