# Supplementary material for: Online Racial/Ethnic Discrimination, Suicidal Ideation, and Alcohol Misuse Among Ethnoracially Minoritized College Students: The Roles of Internalized Racism and Ethnic Identity
Source: J Racial Ethn Health Disparities. 2025 Mar 12;13(3):1874–88. doi: 10.1007/s40615-025-02381-1 (PMC13157410; doi:10.1007/s40615-025-02381-1)
Supplement: Supplementary file 1 — Supplementary file1 (DOCX 17 KB) [file 40615_2025_2381_MOESM1_ESM.docx]

Supplemental Table 1.

| **SI** | **Effect** | **Boot SE** | **Boot LLCI** | **Boot ULCI** |
| --- | --- | --- | --- | --- |
| 16^th^ percentile | .086 | .039 | .030 | .183 |
| 50^th^ percentile | .073 | .028 | .029 | .137 |
| 84^th^ percentile | .067 | .028 | .022 | .130 |
| **Alcohol use** |  |  |  |  |
| 16^th^ percentile | .015 | .018 | -.012 | .058 |
| 50^th^ percentile | .012 | .014 | -.011 | .044 |
| 84^th^ percentile | .011 | .013 | -.011 | .040 |

Conditional indirect effects of individual online racial/ethnic discrimination on SI and alcohol use via internalized racism at different levels of ethnic identity exploration adjusted for sex and race/ethnicity

Supplemental Table 2.

Conditional indirect effects of individual online racial/ethnic discrimination on SI and alcohol use via internalized racism at different levels of ethnic identity commitment adjusted for sex and race/ethnicity

| **SI** | **Effect** | **Boot SE** | **Boot LLCI** | **Boot ULCI** |
| --- | --- | --- | --- | --- |
| 16^th^ percentile | .109 | .046 | .041 | .217 |
| 50^th^ percentile | .068 | .025 | .027 | .125 |
| 84^th^ percentile | .037 | .027 | -.013 | .092 |
| **Alcohol use** |  |  |  |  |
| 16^th^ percentile | .019 | .022 | -.017 | .070 |
| 50^th^ percentile | .112 | .013 | -.011 | .040 |
| 84^th^ percentile | .006 | .008 | -.008 | .024 |

Supplemental Table 3.

Conditional indirect effects of vicarious online racial/ethnic discrimination on SI and alcohol use via internalized racism at different levels of ethnic identity exploration adjusted for sex and race/ethnicity

| **SI** | **Effect** | **Boot SE** | **Boot LLCI** | **Boot ULCI** | |
| --- | --- | --- | --- | --- | --- |
| 16^th^ percentile | .076 | .028 | .030 | .137 |  |
| 50^th^ percentile | .052 | .019 | .020 | .093 |  |
| 84^th^ percentile | .040 | .018 | .010 | .081 |  |
| **Alcohol use** |  |  |  |  |  |
| 16^th^ percentile | .013 | .014 | -.008 | .045 |  |
| 50^th^ percentile | .009 | .009 | -.007 | .030 |  |
| 84^th^ percentile | .007 | .008 | -.006 | .024 |  |

Supplemental Table 4.

Conditional indirect effects of vicarious online racial/ethnic discrimination on SI and alcohol use via internalized racism at different levels of ethnic identity commitment adjusted for sex and race/ethnicity

| **SI** | **Effect** | **Boot SE** | **Boot LLCI** | **Boot ULCI** |
| --- | --- | --- | --- | --- |
| 16^th^ percentile | .110 | .035 | .052 | .189 |
| 50^th^ percentile | .048 | .017 | .021 | .086 |
| 84^th^ percentile | .002 | .014 | -.028 | .030 |
| **Alcohol use** |  |  |  |  |
| 16^th^ percentile | .019 | .018 | -.014 | .057 |
| 50^th^ percentile | .008 | .008 | -.006 | .026 |
| 84^th^ percentile | .000 | .003 | -.007 | .008 |
